# Electrochemical Detection of *ompA* Gene of *C. sakazakii* Based on Glucose-Oxidase-Mimicking Nanotags of Gold-Nanoparticles-Doped Copper Metal-organic Frameworks

**DOI:** 10.3390/s23094396

**Published:** 2023-04-29

**Authors:** Hongyan Zhang, Guiqing Xu, Yuming Chen, Xu Li, Shaopeng Wang, Feihao Jiang, Pengyang Zhan, Chuanfu Lu, Xiaodong Cao, Yongkang Ye, Yunlai Tao

**Affiliations:** 1 School of Food Science and Biological Engineering, Hefei University of Technology, Hefei 230009, Chinaxiaodongcao@hfut.edu.cn (X.C.); 2 Department of Food Science, Xuancheng Campus, Hefei University of Technology, Xuancheng 242000, China; 3 Anhui Institute of Food and Drug Inspection, Hefei 230051, China; tyl3333@sohu.com

**Keywords:** electrochemical biosensor, *C. sakazakii*, gold-nanoparticle-doped copper Metal-organic frameworks, *ompA* gene, glucose-oxidase-mimicking activity

## Abstract

The present work developed an electrochemical genosensor for the detection of virulence outer membrane protein A (*ompA,* tDNA) gene of *Cronobacter sakazakii* (*C. sakazakii*) by exploiting the excellent glucose-oxidase-mimicking activity of copper Metal-organic frameworks (Cu-MOF) doped with gold nanoparticle (AuNPs). The signal nanotags of signal probes (sDNA) that biofunctionalized AuNPs@Cu-MOF (sDNA-AuNPs@Cu-MOF) were designed using an Au-S bond. The biosensor was prepared by immobilization capture probes (cDNA) onto an electrodeposited AuNPs-modified glassy carbon electrode (GCE). AuNPs@Cu-MOF was introduced onto the surface of the GCE via a hybridization reaction between cDNA and tDNA, as well as tDNA and sDNA. Due to the enhanced oxidase-mimicking activity of AuNPs@Cu-MOF to glucose, the biosensor gave a linear range of 1.0 × 10^−15^ to 1.0 × 10^−9^ mol L^−1^ to tDNA with a detection limit (LOD) of 0.42 fmol L^−1^ under optimized conditions using differential pulse voltammetry measurement (DPV). It can be applied in the direct detection of *ompA* gene segments in total DNA extracts from *C. sakazakii* with a broad linear range of 5.4−5.4 × 10^5^ CFU mL^−1^ and a LOD of 0.35 CFU mL^−1^. The biosensor showed good selectivity, fabricating reproducibility and storage stability, and can be used for the detection of *ompA* gene segments in real samples with recovery between 87.5% and 107.3%.

## 1. Introduction

*Cronobacter sakazakii* (*C. sakazakii*), widely found in various kinds of foods, is one of the most dangerous foodborne pathogens for infants and young children [1,2]. It is reported that the mortality rate for the *C. sakazakii*-infected patients is about 40–80% and the pollution source is unclear [3,4]. What is more serious is that the pathogenic mechanism has not been clarified yet. Therefore, rapid prevention, detection, and screening of *C. sakazakii* are particularly important to ensure food safety and quality.

The bacterial culture method for *C. sakazakii* is a traditional method that takes 5 to 7 days to obtain results and cannot meet the needs for rapid detection and screening of bacteria. Some other methods that are classified into protein-based biosensing method [5,6,7] and DNA-based biosensing method [8,9,10,11,12,13,14,15,16] have been promoted and applied in the detection of *C. sakazakii*. Protein-based immunoassays, such as enzyme-linked immunosorbent assays (ELISAs) [5] and lateral flow immunoassays [6], are based on antibody-antigen interactions, which require a certain concentration of targets in samples, and sometimes cannot be applied in the detection at an early infection stage. DNA-based methods including polymerase chain reaction (PCR)-based biosensing methods [8,9], fluorescence biosensing methods [10,11], colorimetric biosensing methods [12,13], and electrochemical biosensing methods [14,15,16] have been intensively promoted and investigated for the detection of *C. sakazakii.* Due to the properties of low cost, ease of fabrication, time-saving, and ease of operation, several kinds of electrochemical biosensors, including amperometric biosensors [14,16] and voltametric biosensors [15], have been fabricated for *C. sakazakii* detection. To enhance the sensitivity of *C. sakazakii* detection, nanomaterials and signal amplification strategies have been used in biosensor construction and *C. sakazakii* detection. For instance, Peng et al. [15] constructed a sensitive electrochemical aptasensor using the nanomaterial of methylene blue (MB)-anchored graphene oxide (GO). By using the large surface area and abundant recognition sites of GO, the electrochemical oxidation signal MB was significantly enhanced. The fabricated biosensor showed a low detection limit of 7 CFU mL^−1^ (*S*/*N* = 3). 

Metal-organic frameworks (MOF) are a type of inorganic-organic hybrid nanomaterial, in which the metal ions act as the connectors and the organic ligands as the linkers [17]. It has been widely used in energy storage [18,19,20], gas storage and separation [21], drug delivery [22], chemical sensors [23], and biosensors [24] due to the properties of large surface area, tunable structure, good catalytic activity, and biocompatibility. Cu-MOF as a member of the MOF family has been commonly used in fabricating chemical sensors [25,26] and biosensors [27,28] by utilizing its good conductivity, large surface area, excellent catalytic ability, and ease of surface modification. Research shows that Cu-based MOF have excellent mimic oxidase activities to various molecules, such as H_2_O_2_ [25,29,30], ascorbate [26], and glucose [30,31,32,33].

**Figure 1:**
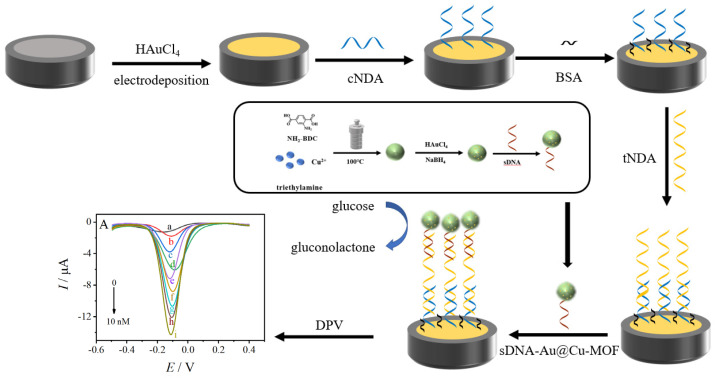
A schematic illustration of the fabrication of the biosensor.

Gold nanoparticles (AuNPs) and AuNP-based nanomaterials have been proven to be excellent electrocatalysts to glucose [34,35]. In the present work, we constructed a DNA biosensor for the virulence outer membrane protein A (*ompA*) gene of *C. sakazakii* based on glucose-oxidase-mimicking AuNPs-doped Cu-MOF (AuNPs@Cu-MOF). As shown in Figure 1, Cu-MOF was prepared using 2-aminoterephthalic acid (NH_2_-BDC) as a ligand, Cu(II) as a metal center, and triethylamine as a structure regulator. The prepared Cu-MOF was then used as carriers for doping AuNPs to synthesize AuNPs@Cu-MOF. Then signal probe DNA (sDNA) was immobilized on AuNPs@Cu-MOF via Au-S bond, forming signal nanotags of sDNA-AuNPs@Cu-MOF. The biosensor was constructed on a glassy carbon electrode modified in sequence with electrodeposited AuNPs, capture probe DNA (cDNA) for target *ompA* gene (tDNA), and BSA. Subsequently, tDNA was hybridized with cDNA and sDNA-AuNPs@Cu-MOF, bringing AuNPs@Cu-MOF to the surface of the modified electrode. Owing to the excellent mimic oxidase activity of AuNPs@Cu-MOF to glucose, the biosensor exhibited good analytical performance to target the *ompA* gene and *C. sakazakii* with a detection limit of 0.42 fmol L^−1^ and 0.35 CFU mL^−1^.

## 2. Experimental Section

### 2.1. Materials

Polyvinylpyrrolidone K30 (PVP), N,N-dimethylformamide (DMF), ethanol, glucose, triethylamine, Tris-(2-carboxyethyl) phosphine (TCEP), 2-aminoterephthalic acid (NH_2_-BDC, 99%), and bovine serum albumin (BSA, 98%) were purchased from Aladdin Industrial Co., Ltd. (Shanghai, China). Chloroauric acid (HAuCl_4_), copper nitrate-trihydrate (Cu(NO_3_)_2_·3H_2_O), and ethylenediaminetetraacetic acid disodium salt dihydrate (C_10_H_14_N_2_Na_2_O_8_·2H_2_O) were obtained from National Medicine Group Shanghai Chemical Reagent Co., Ltd. (Shanghai, China). TAE (50×) and Taq PCR Mix (2×) were purchased from Shanghai Sangon Biotechnology Co., Ltd. (Shanghai, China). All other chemicals were of analytical grade and used without further treatment. The buffers used in this work were as follows: (1) DNA stock buffer (pH 7.4): a mixture of 10 mmol L^−1^ Tris-HCl and 1.0 mmol L^−1^ EDTA; (2) DNA immobilization buffer (pH 7.4): a mixture of 10 mmol L^−1^ Tris-HCl, 1.0 mmol L^−1^ EDTA, 1.0 mmol L^−1^ TCEP, 100 mmol L^−1^ NaCl, and 1.0 mmol L^−1^ MgCl_2_; (3) DNA hybridization buffer (pH 7.4): 10 mmol L^−1^ Tris-HCl, 1.0 mmol L^−1^ EDTA, 100 mmol L^−1^ NaCl, and 1.0 mmol L^−1^ MgCl_2_; (4) electrochemical detection buffer (pH 7.0): 0.1 mol L^−1^ phosphate-buffered solution (PBS) containing 6.0 mmol L^−1^ glucose. All the solutions were freshly prepared using ultrapure water (resistivity ≥ 18 MΩ cm).

All DNA sequences purchased from Shanghai Sangon Biotechnology Co., Ltd. (Shanghai, China) are listed in Table 1. Strains were provided by the Technology Center of Hefei Customs District. The total DNA of cultivated strains was extracted by using Bacterial Genomic DNA Rapid Extraction Kit (071011 M, 48 T) which was from Guangzhou Double Helix Gene Technology Co., Ltd. (Guangzhou, China).

### 2.2. Apparatus

Scanning electron microscopy (SEM, Gemini 300, Zeiss, Jena, Germany) was carried out at an accelerating voltage of 3.0 kV, and X-ray photoelectron spectroscopy (XPS) measurements were conducted on an ESCALAB 250XI (Thermo Fisher Scientific, Waltham, MA, USA). The PCR reaction was conducted on TC-96/T/H PCR Amplifier (Hangzhou Bioer Technology Co., Ltd., Hangzhou, China). All the electrochemical measurements in terms of cyclic voltammetry (CV), electrochemical impedance spectroscopy (EIS), and differential pulse voltammetry (DPV) were performed on a CHI660D electrochemical workstation (Chenhua Instruments Inc., Shanghai, China) with a traditional three-electrode configuration. While performing measurements, a modified glassy carbon electrode (GCE), a platinum wire, and an Ag/AgCl (sat. KCl) electrode were used as the working, counter, and reference electrodes, respectively.

### 2.3. Synthesis of AuNPs@Cu-MOF

The Cu-MOF was prepared according to the literature [32,33] with some revisions. Typically, solution A was prepared by dissolving 0.2 g PVP in a mixture containing 4 mL DMF and 4 mL ethanol, and solution B was prepared by mixing 18.1 mg Cu(NO_3_)_2_·3H_2_O and 5.4 mg NH_2_−BDC in 4 mL DMF. Then, solution B was added into solution A, and ultrasonicated for 5 min, followed by the addition of 2 μL triethylamine. The mixture was thoroughly mixed and then transferred into a Teflon-lined autoclave. The hydrothermal reaction was performed at 100 °C for 6 h. After cooling down to room temperature, the precipitate was collected, dissolved in 20 mL of DMF again, and reacted at 100 °C in the Teflon-lined autoclave for a further 8 h. Finally, the obtained Cu-MOF were centrifuged and freeze-dried for further use.

AuNPs@Cu-MOF was prepared by in situ reduction of AuCl_4_^−^ on the surface of Cu-MOF. Briefly, Cu-MOF (10 mg) was first dispersed in 5 mL of deionized water. Then, 20 μL of 1% HAuCl_4_ and 2 mL of 2 mmol L^−1^ NaBH_4_ were added into the Cu-MOF dispersion with gentle shaking. The reaction was then performed at 4 °C for 1 h with continuous stirring. After centrifugation and washing several times, the obtained AuNPs@Cu-MOF was finally redispersed in a 2 mL DNA immobilization buffer.

### 2.4. Preparation of sDNA-Functionalized AuNPs@Cu-MOF Signal Probes

sDNA-functionalized AuNPs@Cu-MOF (sDNA-AuNPs@Cu-MOF) was prepared through the reaction between Au and −SH. A volume of 100 μL of 10 μM thiolated sDNA was added into the dispersion of AuNPs@Cu-MOF, followed by shaking for 12 h at 4 °C. The obtained sDNA-AuNPs@Cu-MOF was centrifuged and washed several times and then dispersed in DNA storage buffer (2 mL) and stored at 4 °C.

### 2.5. Fabrication of the ompA Biosensor

The *ompA* biosensor was fabricated on the GCE surface (Figure 1). Before modification, bare GCE was polished with 1.0, 0.3, and 0.05 μm α-Al_2_O_3_ slurry, ultrasonicated in ethanol and water in sequence, and finally dried under nitrogen flow to obtain a clean, mirror-like surface. Firstly, the pretreated GCE was immersed in 0.1 mol L^−1^ KNO_3_ (5 mL) solution containing 3.0 mmol L^−1^ HAuCl_4_. The electrode was then operated at a constant potential of −0.2 V for 70 s to obtain electrodeposited AuNPs-modified GCE (Au/GCE). Secondly, 10 μL of 1 μmol L^−1^ cDNA was cast onto the Au/GCE and then capped with a centrifuge tube on top and kept at 4 °C for 12 h. Then, the electrode was rinsed with 0.1% SDS solution and Tris-HCl solution (10 mmol L^−1^, pH = 7.4) to remove the unbound capture probe. The modified electrode was denoted as cDNA/Au/GCE. Finally, the *ompA* biosensor was accomplished by immersing cDNA/Au/GCE in 1% BSA for 30 min to eliminate nonspecific adsorption.

### 2.6. Preparation of DNA Samples and PCR of the ompA Gene Products

The preparation of DNA samples and PCR products of *ompA* gene segments was according to our previous work [14,36]. First, *C. sakazakii* solution with various concentrations was prepared according to previous work with slight modification [14]. Briefly, *C. sakazakii* was activated in sterilized LB broth for 24 h at 37 °C with a rotation speed of 100 rpm. The activated strains (5 μL) were inoculated in LB broth and cultivated for 12 h at 37 °C with a rotation speed of 100 rpm. The solution was then diluted with sterilized 0.9% NaCl to different concentration gradients, and 50 μL of each bacterial solution with various concentrations was inoculated on a solid medium for spreading plates. After 12 h of cultivation at 37 °C, the bacterial solution concentration was determined by counting. Second, total DNA in *C. sakazakii* was extracted using Bacterial Genomic DNA Rapid Extraction Kit. The original bacterial solution (1 mL) was centrifuged at 10,000 rpm for 5 min at 4 °C. The precipitate was collected and added to a sterile 0.9% NaCl aqueous solution (500 μL). The precipitate was well dispersed in the mixture by vortex mixing. The mixture was centrifuged at 10,000 rpm for 5 min to collect the precipitate. Afterwards, 200 μL of DNA extraction solution was added to the precipitate, followed by vigorous vortexing. It was then heated at 100 °C for 10 min and followed by rapid cooling in the refrigerator for another 10 min. The mixture was centrifuged at 10,000 rpm for 3 min, and the supernatant was collected and stored at 4 °C.

The DNA extraction samples of *C. sakazakii-contaminated* infant formula powder were prepared according to Zhao’s method [34]. Three actual samples were prepared by adding cultured *C. sakazakii* (5.4 × 10^1^, 5.4 × 10^2^, and 5.4 × 10^3^ CFU mL^−1^) into infant formula powder (1.0 g) which was dissolved in buffer peptone water (10.0 mL). The solution was thoroughly mixed, followed by removing proteins and fats using chloroform. Typically, after the addition of 1.0 mL chloroform, the contaminated suspension was kept shaking for 1 min. The resultant mixture was centrifuged at 3000 rpm for 5 min at room temperature. The supernatant was then collected and transferred into a centrifugal tube, followed by centrifugation at 12,000 rpm for another 5 min. The precipitation was then collected and used for total DNA extraction according to the previous procedures. The prepared real samples were stored at 4 °C in a refrigerator for direct electrochemical detection and PCR.

The PCR amplification was carried out in a reaction system containing 25.0 μL of 2 × Taq PCR Mix, 5.0 μL of total DNA extraction with different concentrations as the template DNA, 1.0 μL of 10.0 μM Primer F, 1.0 μL of 10.0 μM Primer R, and 18.0 μL of double distilled H_2_O. The PCR amplification procedures were as follows: pre-denaturation at 95 °C for 5 min, 30 amplification cycles (denaturation at 95 °C for 30 s, annealing at 58 °C for 40 s, and extension at 72 °C for 40 s), and finally, extension at 72 °C for 5 min. The obtained PCR product was stored at 4 °C in the refrigerator. Agarose gel electrophoresis was used to verify the PCR products. The measurement was performed on a 1% agarose gel at 120 V for 40 min in 1 × TAE buffer, and the electrophoresis bands were observed under ultraviolet light.

### 2.7. Electrochemical Measurement

Cyclic voltammetry (CV) was used to characterize the catalytic ability of Cu-MOF and AuNPs@Cu-MOF to glucose. The experiments were performed in phosphate buffer solution (PBS, 0.1 M, pH 7.0) containing 5.0 mM glucose within a potential window of −0.8~0.4 V at a scan rate of 50 mV s^−1^.

CV and electrochemical impedance spectroscopy (EIS) were adopted for monitoring the fabrication process of GCE. The CV experiments were carried out in 0.1 M KCl containing 5.0 mM [Fe(CN)_6_]^3−/4−^ between the potential window −0.5 V and 0.6 V with a scan rate of 50 mV s^−1^. The EIS measurements were performed in 0.1 M KCl containing 5.0 mM [Fe(CN)_6_]^3−/4−^ in a frequency range of 0.1~100 kHz.

The analytical performance of our constructed biosensor was evaluated by differential pulse voltammetry (DPV). As shown in Figure 1, the fabricated *ompA* biosensor (BSA-blocked cDNA/Au/GCE) was incubated in hybridized buffer containing various concentrations of the *ompA* target. The hybridization reaction was carried out at 37 °C for 60 min. After rinsed with Tris-HCl solution (10 mmol L^−1^, pH 7.4), the obtained tDNA/cDNA/Au/GCE was incubated in sDNA-AuNPs@Cu-MOF for 90 min at 37 °C to undergo the hybridization reaction between tDNA and sDNA. The resultant modified electrode also rinsed with Tris-HCl solution (10 mmol L^−1^, pH 7.4) was named as sDNA-Au@Cu-MOF/tDNA/cDNA/Au/GCE. The DPV measurements were performed in a potential range from −0.5 to 0.4 V in 0.1 mol L^−1^ phosphate buffer solution (pH = 7.0) containing 6.0 mM glucose. The DPV parameters were set as follows: a pulse amplitude of 50 mV and a pulse width of 0.05 s.

## 3. Results and Discussion

### 3.1. Characteristics of Cu-MOF and AuNPs@Cu-MOF

**Figure 2:**
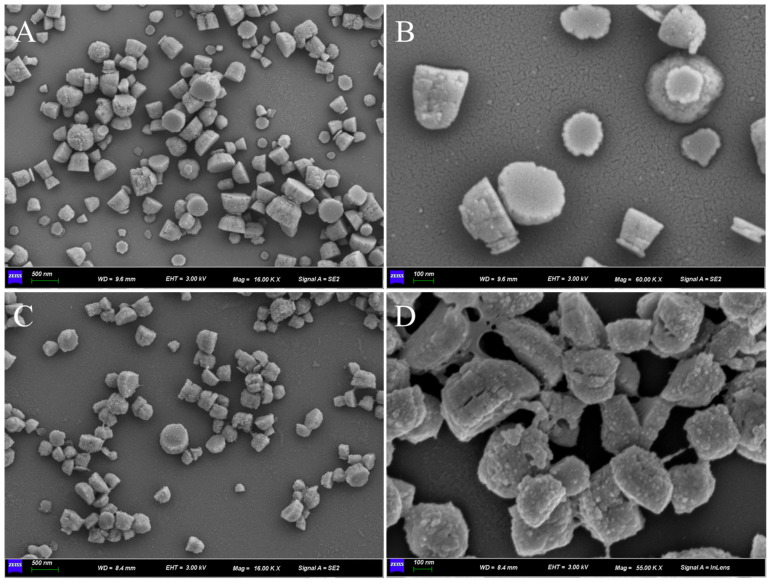
SEM images of Cu-MOF and AuNPs@Cu-MOF obtained with different magnifications. Cu-MOF: (**A**) and (**B**) and AuNPs@Cu-MOF: (**C**) and (**D**).

The morphology of Cu-MOF and AuNPs@Cu-MOF was characterized by SEM, and the images are shown in Figure 2. As shown in Figure 2A,B, the particle size of Cu-MOF ranges from 150 to 500 nm. The particles appear as cup-like structures. Compared with Cu-MOF, after the immobilization of chemically reduced AuNPs, the surface of AuNPs@Cu-MOF (Figure 2C,D) becomes rougher. This indicates the successful loading of AuNPs on Cu-MOF. Moreover, the particle size of AuNPs@Cu-MOF also ranges from 150 to 500 nm, and the shape of AuNPs@Cu-MOF is still in a cup-like structure. More importantly, from the SEM images of Cu-MOF and AuNPs@Cu-MOF, the background is clear, demonstrating the stable structure possessed by these materials.

**Figure 3:**
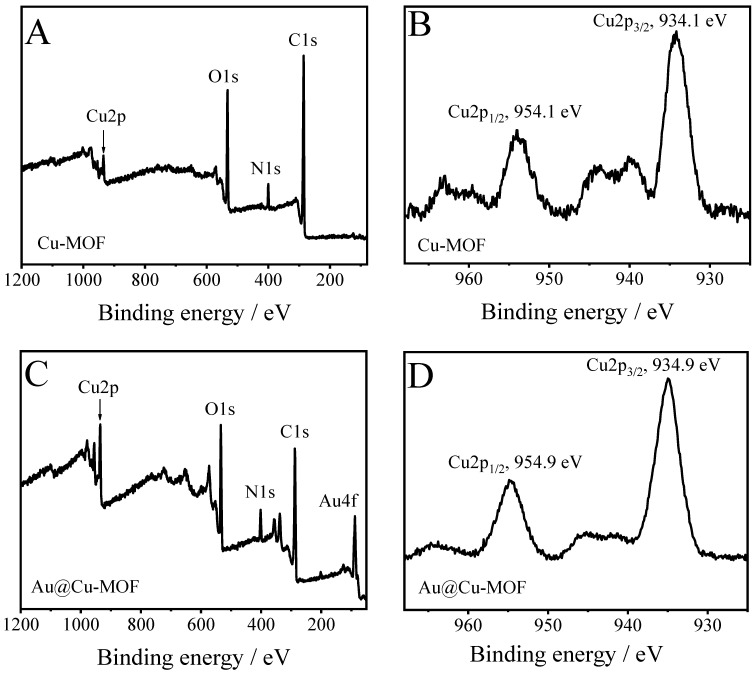
(**A**) XPS survey spectrum of Cu-MOF, (**B**) Cu2p region in Cu-MOF, (**C**) XPS survey spectrum of AuNPs@Cu-MOF, and (**D**) Cu2p region in AuNPs@Cu-MOF.

The elemental composition of Cu-MOF and AuNPs@Cu-MOF was characterized by using XPS. As displayed in Figure 3A, the survey spectrum of Cu-MOF gives the main peaks located at 934.2, 532.1, 400.0, and 285.3 eV that are ascribed to Cu2p [32], O1s [37], N1s [37], and C1s [37], respectively. The Cu2p spectrum in Cu-MOF (Figure 3B) shows that the binding energies of Cu^2+^ 2p_1/2_ and Cu^2+^ 2p_3/2_ are about 954.1 and 934.1 eV, respectively. From the XPS survey of AuNPs@Cu-MOF (Figure 3C), apart from the peaks of Cu2p, O1s, N1s, and C1s, a new peak at 87.7 eV can be found. This peak is assigned to Au4f [14]. In addition, the binding energies of Cu^2+^ 2p_1/2_ and Cu^2+^ 2p_3/2_ of the Cu2p spectrum in AuNPs@Cu-MOF (Figure 3D) are about 954.9 and 934.9 eV, respectively. The immerged peak of Au4f in the AuNPs@Cu-MOF XPS survey indicates the loading of AuNPs on Cu-MOF, which is in accordance with the result obtained from SEM characterization. Compared with the binding energy of Cu^2+^ 2p_1/2_ and 2p_3/2_ in Cu-MOF, the positive shift of Cu^2+^ 2p_1/2_ and 2p_3/2_ in AuNPs@Cu-MOF shows that the electron was away from divalent Cu in the structure of AuNPs@Cu-MOF. This may cause the valence of Cu in AuNPs@Cu-MOF to be higher than that in Cu-MOF. Moreover, no peaks attributed to Cu^0^ and Cu^+^ in Cu-MOF and AuNPs@Cu-MOF were observed. This indicates that Cu-MOF was formed through the reaction between Cu^2+^ and organic ligands, and no structural damage of Cu-MOF occurred during the reduction of AuCl^4−^ to Au^0^ using NaBH_4_ as a reducing agent.

**Figure 4:**
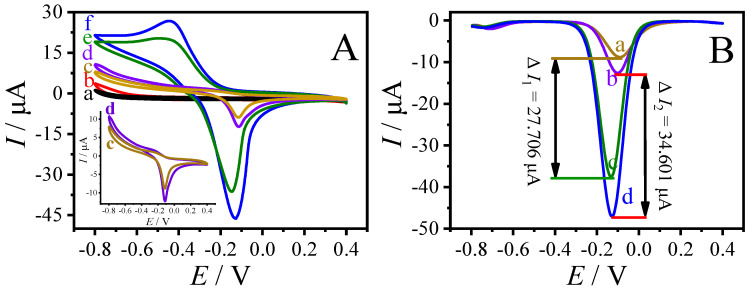
(**A**) CV curves obtained on different electrodes in 0.1 M pH 7.0 PBS with (b, e, and f) or without (a, c, and d) 5 mM glucose at a scan rate of 50 mV s^−1^. (a) and (b) were at bare GCE, (c) and (e) were at Cu-MOF/GCE, and (d) and (f) were at AuNPs@Cu-MOF/GCE. (**B**) DPV curves obtained at Cu-MOF/GCE (a and c) and AuNPs@Cu-MOF/GCE (b and d) in 0.1 M pH 7.0 PBS with (c and d) or without (a and b) 5 mM glucose.

The mimetic glucose oxidase activity of Cu-MOF and AuNPs@Cu-MOF was investigated and compared by CV and DPV. Figure 4A shows the CV curves obtained on different electrodes in 0.1 M pH 7.0 PBS with or without 5 mM glucose. As shown in the figure, no redox peaks were observed within the sweeping potential window of −0.8~0.4 V in the presence (curve b) or absence (curve a) of 5 mM glucose. In the absence of glucose, an oxidation peak was recorded at about −0.11 V and a reduction peak at about −0.22 V on Cu-MOF/GCE and AuNPs@Cu-MOF/GCE. In the presence of 5 mM glucose, however, the redox peak currents at −0.11 V and −0.22 V were dramatically increased. Furthermore, the peak current values of oxidation and reduction peaks were higher at AuNPs@Cu-MOF/GCE than those at Cu-MOF/GCE. The electrocatalytic ability of Cu-MOF and AuNPs@Cu-MOF to glucose was also evaluated and compared by DPV (Figure 4B) in a potential range between −0.8 and 0.4 V. As can be seen from the figure, the absolute values of peak current on AuNPs@Cu-MOF/GCE and Cu-MOF/GCE were calculated to be 34.601 (Δ*I*_2_) and 27.706 μA (Δ*I*_1_), respectively. The electrochemical reaction that occurred on the modified electrode may follow the reaction steps given below:

Cu(II)-MOF − e → Cu(III)-MOF(1)

Cu(III)-MOF + glucose → Cu(II)-MOF + gluconolactone(2)

It can be inferred from the above reaction mechanism that the amplified signal on Cu-MOF/GCE and AuNPs@Cu-MOF/GCE is due to the electrocatalytic ability of Cu(II) anchored in MOFs to glucose [38,39]. The enhanced catalytic ability of AuNPs@Cu-MOF may result from the synergistic effect of AuNPs and Cu-MOF [25]. It is worth mentioning that the higher valence of Cu in AuNPs@Cu-MOF than that in Cu-MOF may also be the reason for its enhanced oxidation ability to glucose.

### 3.2. Electrochemical Characterization of Biosensor Fabrication

**Figure 5:**
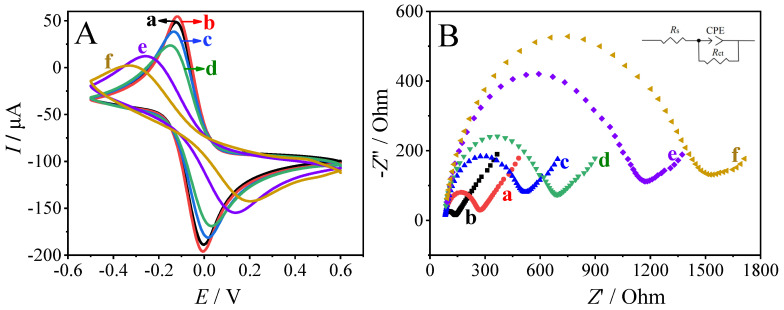
CVs (**A**) and EIS plots (**B**) recorded on bare GCE (a), Au/GCE (b), cDNA/Au/GCE (c), BSA blocked cDNA/Au/GCE (d), tDNA/BSA/cDNA/Au/GCE (e), and sDNA-AuNPs@Cu-MOF/tDNA/cDNA/Au/GCE (f) in 0.1 M KCl containing 5 mM [Fe(CN)6]^3−/4−^.

The step-by-step assembly of the biosensor was characterized by CV and EIS in 0.1 M KCl containing 5.0 mM [Fe(CN)6]^3−/4−^. As exhibited in Figure 5A, a pair of well-defined redox peaks were observed on bare GCE and different modified GCEs. The absolute values of the redox peaks recorded on Au/GCE (Figure 5A, curve b) were increased compared with those recorded on bare GCE (Figure 5A, curve a), indicating the electron facilitation of the electrodeposited AuNPs. Afterwards, the absolute values of the redox peak currents decreased step by step with the immobilization of cDNA (Figure 5A, curve c), BSA (Figure 5A, curve d), tDNA (Figure 5A, curve e), and sDNA-AuNPs@Cu-MOF (Figure 5A, curve f) on Au/GCE, indicating the inhibited diffusion of [Fe(CN)_6_]^3−/4−^ redox probe. In addition, the variation trend of charge transfer resistance (*R*_ct_) of GCE and different modified GCEs analyzed by EIS (Figure 5B) was in accordance with the CV result. As is clearly shown in Fig. 5B, bare GCE showed a small semicircle with an *R*_ct_ of 217.7 Ω (Figure 5B, curve a). The obtained *R*_ct_ on Au/GCE was significantly decreased to 127.3 Ω (Figure 5B, curve b), owing to fast electron transfer on Au/GCE. After stepwise modification of cDNA (Figure 5B, curve c), BSA (Figure 5B, curve d), tDNA (Figure 5B, curve e), and sDNA-AuNPs@Cu-MOF (Figure 5B, curve f) on Au/GCE, the *R*_ct_ increased step by step to 446.3, 600.6, 1075.0, and 1398.0 Ω. This demonstrates the inhibited diffusion of [Fe(CN)6]^3−/4−^ redox probe in 0.1 M KCl solution. Both the CV and EIS characterizations show the successful fabrication of the *ompA* gene-sensor and its ability to bind target probe and signal probe via DNA hybridization.

### 3.3. Analytical Performance of the Biosensor

**Figure 6:**
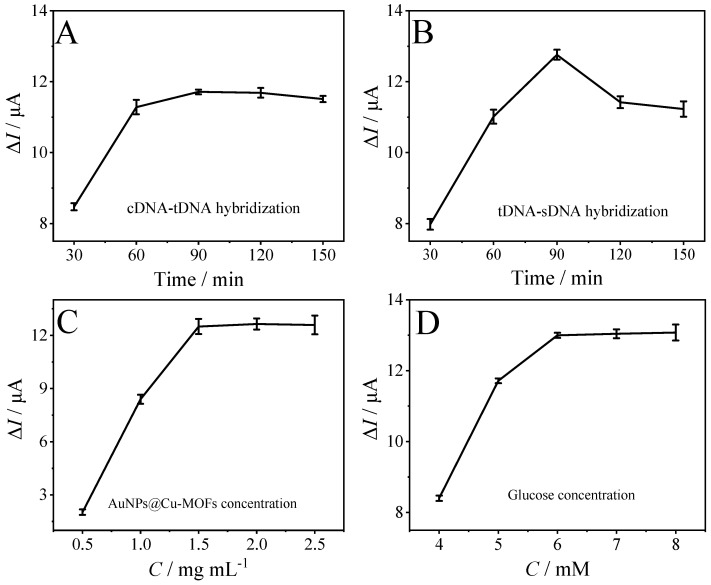
Parameter optimization of the biosensor. (**A**) Effect of cDNA and tDNA hybridization time, (**B**) effect of tDNA and sDNA hybridization time, (**C**) effect of Au@Cu-MOF concentration, and (**D**) effect of glucose concentration. The concentration of tDNA was 10 nM.

The analytical performance of the biosensor for detecting the *ompA* gene was investigated by using the DPV method. Before conducting DPV measurements, a number of factors, including the hybridization time between cDNA and tDNA, hybridization time between tDNA and sDNA, the concentration of AuNPs@Cu-MOF, and the concentration of glucose in the electrochemical detection buffer, were optimized. The relationship between the change of peak current (Δ*I*, Δ*I* = *I*_0_ − *I*_1_, where *I*_0_ and *I*_1_ were the current responses of the biosensor to 0 and 10 nmol L^−1^ tDNA, respectively) and different parameters was shown in Figure 6. As displayed in the figure, it is easy to conclude that the optimal conditions for detecting the *ompA* gene were as 90 min for cDNA and tDNA hybridization, 90 min for tDNA and sDNA hybridization, 1.5 mg mL^−1^ of AuNPs@Cu-MOF, and 6 mM glucose in electrochemical detection buffer.

**Figure 7:**
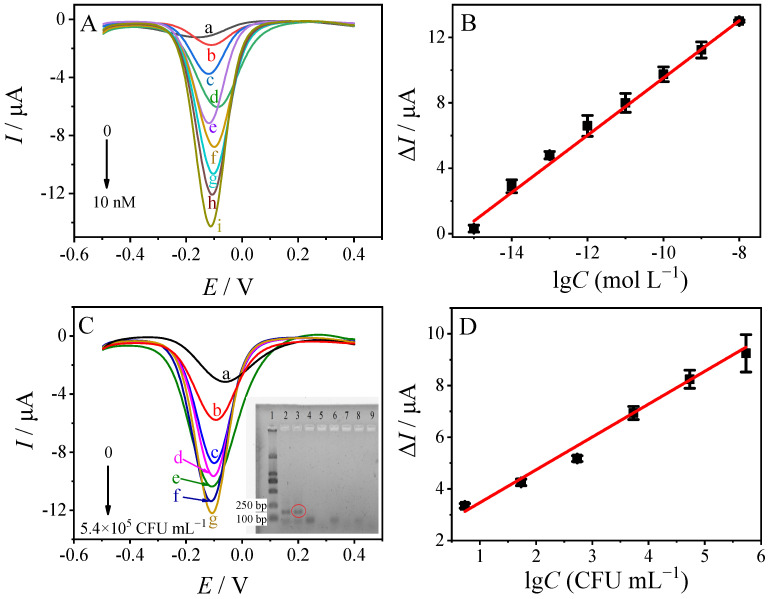
(**A**) DPV responses of the biosensor to different tDNA concentrations in 0.1 M pH 7.0 PBS contained 6.0 mM glucose. From top to bottom: (a) 0 M, (b) 1 fM, (c) 10 fM, (d) 100 fM, (e) 1 pM, (f) 10 pM, (g) 100 pM, (h) 1 nM, and (i) 10 nM. Inset: (**B**) The calibration curve of Δ*I* vs. the logarithm of tDNA in the range of 1 fM~10 nM. (**C**) The DPV responses of the biosensor towards various concentrations of *ompA* gene segments in total DNA extracted from different concentrations of *Cronobacter sakazakii*. From top to bottom are the curves of detecting 0, 5.4, 54, 5.4 × 10^2^, 5.4 × 10^3^, 5.4 × 10^4^, 5.4 × 10^5^ CFU mL^−1^. Inset: agarose gel electrophoresis for marker (lane 1), *ompA* gene PCR products of 5.4 × 10^5^, 5.4 × 10^4^, 5.4 × 10^3^, 5.4 × 10^2^, 54, and 5.4 CFU mL^−1^ (from lanes 2 to 7, respectively). (**D**) The linear relationship between Δ*I* and the logarithm concentration of *Cronobacter sakazakii*.

The biosensor was then applied in the detection target *ompA* gene under optimal conditions, and the DPV responses to various concentrations of *ompA* are displayed in Figure 7A. As clearly shown in the figure, the DPV response increases with the increasing *ompA* concentration. In this biosensing system, cDNA, tDNA, and sDNA-AuNPs@Cu-MOF can form a sandwich-typed structure of cDNA-tDNA-sDNA-AuNPs@Cu-MOF. As a result, with the increasing concentration of tDNA, more sDNA-AuNPs@Cu-MOF can react with tDNA, leading to the increased electrocatalytic signal of glucose. The DPV response of Δ*I* exhibited a linear relationship with the logarithm tDNA concentration in a range of 1 fmol L^−1^~10 nmol L^−1^ (Figure 7B). The linear regression equation was written as Δ*I* (μA) = 1.749 lg*C* (mol⋅L^−1^) + 27.01 (*R* = 0.997, Equation (1)) with a detection limit of (LOD) 0.42 fmol L^−1^ (*S*/*N* = 3).

### 3.4. Detection of ompA Gene Segments Extracted from Cronobacter sakazakii

The analytical performance of the fabricated biosensor was also investigated and evaluated by using it to directly detect *ompA* gene segments in total DNA extracted from *C. sakazakii*. The *ompA* gene segments extracted from *C. sakazakii* were amplified with PCR, and the products were confirmed with agarose gel electrophoresis (Figure 7C, inset). In this figure, lanes 2 to 7 were the PCR products of various concentrations of *C. sakazakii* (5.4 × 10^5^, 5.4 × 10^4^, 5.4 × 10^3^, 5.4 × 10^2^, 54, and 5.4 CFU mL^−1^, respectively). Compared with the marker (lane 1), the band at between 100 bp and 250 bp in lanes 2 and 3 is related to PCR products of *ompA* gene segments. The DPV responses on the biosensor for *ompA* segments in total DNA extracted from various concentrations of *C. sakazakii* are shown in Figure 7C. As shown in the figure, the recorded DPV response on the biosensor varied with the varying concentration of *C. sakazakii*. It is obvious that the DPV response increased with the increasing concentration of *C. sakazakii*. A good linear relationship between Δ*I* and the logarithm of the *C. sakazakii* concentration was obtained in a range of 5.4–5.4 × 10^5^ CFU mL^−1^ with an equation of Δ*I* (μA) = 1.271 lg*C* (CFU mL^−1^) + 2.196 (*R* = 0.996, Equation (2)). The LOD was calculated to be 0.35 CFU mL^−1^ (*S*/*N* = 3).

The analytical performance of the fabricated biosensor was compared with other reported biosensors for the detection of *C. sakazakii*, and the results are listed in Table 2. As shown in the table, the detection linear range of *C. sakazakii* of our biosensor is broader than most of the electrochemical listed biosensors [12,13,14,15,16], and LOD is the lowest among these biosensors.

### 3.5. Selectivity, Reproducibility, and Stability of Biosensors

**Figure 8:**
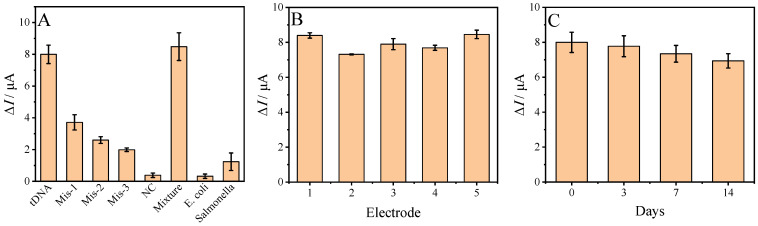
(**A**) Selectivity of the biosensor toward different DNA sequences, (**B**) fabrication reproducibility of the biosensor, and (**C**) storage stability of the biosensor.

The specificity of the fabricated biosensor was evaluated with the DPV method by using the biosensor in detecting various DNA sequences. These sequences were tDNA (10 pmol L^−1^), noncomplementary DNA (NC, 100 pmol L^−1^), single-base mismatched DNA (Mis-1, 100 pmol L^−1^), double-base mismatched DNA (Mis-2, 100 pmol L^−1^), three-base mismatched DNA (Mis-3, 100 pmol L^−1^), and DNA extracts from Escherichia coli (*E*. *coli*, 1.0 × 10^3^ CFU mL^−1^) and Salmonella (1.0 × 10^3^ CFU mL^−1^). The DPV responses to these sequences are shown in histogram style in Figure 8A. The fabricated biosensor gave a strong response to tDNA in the detection buffer and tDNA mixed with other DNA sequences (Figure 8A, column Mixture). The calculated Δ*I* to tDNA and tDNA in the mixture were 7.996 ± 0.580 μA and 8.483 ± 0.871 μA, respectively. This shows that the difference in DPV response on the biosensor for tDNA and tDNA in a mixture was little. Compared to the tDNA signal, the DPV response decreased dramatically to other DNA sequences and DNA extracts from *E*. *coli* and *Salmonella*. These results indicate that the fabricated biosensor has good selectivity for target *ompA*.

The fabrication reproducibility of the electrochemical DNA sensor was also investigated before the application in the detection of *ompA* gene segments in real samples. Five GCEs were used to fabricate five biosensors for the detection of a 10 pM target, and Δ*I* obtained on each biosensor is shown in Figure 8B. A relative standard deviation of 4.81% was obtained within these five constructed biosensors, showing good reproducibility of the biosensor. The storage stability of the fabricated biosensor was also investigated (Figure 8C). After 3, 7, and 14 days of storage, the obtained Δ*I* were 97.2%, 91.8%, and 86.7% of the initial signal (0 day). This suggests that the biosensor has satisfactory storage stability.

### 3.6. Application in Direct Detection of C. sakazakii in Real Samples

Finally, the biosensor was applied in the detection of *C. sakazakii* in real samples. Three pretreated samples of infant formula contaminated with different concentrations (5.4 × 10^1^, 5.4 × 10^2^, and 5.4 × 10^3^ CFU mL^−1^) of *C. sakazakii*. The biosensor was then used for the detection of *ompA* gene segments in total DNA extracts. The detected concentrations of these samples were calculated according to Equation (2), and the results are listed in Table 3. As shown in the table, the recovery of these samples ranged from 87.5% ± 7.6% to 107.3% ± 3.7%, which shows that the constructed sensor has applicability in the detection of the *ompA* gene in real samples.

## 4. Conclusions

In this research work, we designed an electrochemical DNA sensor for the selective detection of the *ompA* gene of *C. sakazakii* by exploiting the excellent electrocatalytic activity of AuNPs@Cu-MOF to glucose. The biosensor showed good linearity from 1 fmol L^−1^ to 10 nmol L^−1^ to target the *ompA* gene with a LOD of 0.42 fmol L^−1^. In addition, the biosensor can be used for direct detection of *ompA* gene fragments in total DNA extracts from *C. sakazakii* with a LOD as low as 0.35 CFU mL^−1^. The constructed electrochemical biosensor possessed good selectivity, fabrication reproducibility and storage stability, which can be applied in the detection of the *ompA* gene in actual samples with good credibility.

## Figures and Tables

**Table 1:** Nucleotide sequences used in the present work.

DNA Sample	Sequences
cDNA	5′-SH-AGC ATG CCG-3′
tDNA	5′-CGC TCG TCC GGA CAA CGG CAT GCT-3′
sDNA	5′-SH-TTG TCC GGA CGA GCG-3′
Mis-1	5′-CGC TCG TCC GGA CAA CGG C***T***T GCT-3′
Mis-2	5′-CGC TCG T***G***C GGA CAA CGG C***T***T GCT-3′
Mis-3	5′-CGC TCG T***G***C GGA CAA C***C***G C***T***T GCT-3′
NC	5′-ACT AGC CTT CCT TGG GAA GTA CTC-3′
Primer F	5′-CAT TGG TGA CGC GCA GAC T-3′
Primer R	5′-TGG ACG GGA TAC CTT TGG-3′

**Table 2:** Comparison of the different detection methods.

Materials/ Nanomaterials	Analytical Method	Linear Range (CFU mL^−1^)	Detection Limit (CFU mL^−1^)	Refs.
AuNPs	colorimetric	7.1 × 10^3^~7.1 × 10^7^	-	[12]
G-rich DNA probes formed DNAzyme	colorimetric	2~1.2 × 10^3^	1.2	[13]
BQDs-AuNPs	i-t	7.8 × 10^0^~7.8 × 10^6^	2.6	[14]
MB-anchored GO	DPV	1.0 × 10^2^~1.0 × 10^7^	73	[15]
G-quadruplex DNAzyme	i-t	3.84 × 10^4^~2.4 × 10^7^	501	[16]
Au@Cu-MOF	DPV	5.4 × 10^0^~5.4 × 10^5^	0.35	This work

**Table 3:** Detection of *C. sakazakii* in real samples (*N* = 3).

Sample	Add (CFU mL^−1^)	Found (CFU mL^−1^)	Recovery (%)
1	5.4 × 10^1^	57.9 ± 2.1	107.3 ± 3.7
2	5.4 × 10^2^	572.4 ± 54.8	105.9 ± 9.5
3	5.4 × 10^3^	4824.7 ± 584	87.5 ± 7.6

## Data Availability

Not applicable.
